# Pattern of prefrontal cortical activation and network revealed by task-based and resting-state fNIRS in Parkinson’s disease’s patients with overactive bladder symptoms

**DOI:** 10.3389/fnins.2023.1142741

**Published:** 2023-03-30

**Authors:** Miaomiao Hou, Xiaowei Mao, Yarong Wei, Jiali Wang, Yu Zhang, Chen Qi, Lu Song, Ying Wan, Zhihua Liu, Jing Gan, Zhenguo Liu

**Affiliations:** ^1^Department of Neurology, Xinhua Hospital Affiliated to Shanghai Jiao Tong University School of Medicine, Shanghai, China; ^2^Department of Neurology, The First Affiliated Hospital of Naval Medical University, Shanghai, China; ^3^Department of Neurosurgery, The First Affiliated Hospital of Naval Medical University, Shanghai, China

**Keywords:** Parkinson’s disease, overactive bladder, prefrontal cortex, fNIRS, resting state, verbal fluency task

## Abstract

**Background:**

Overactive bladder (OAB) symptoms are common in Parkinson’s disease (PD), and negatively contribute to the quality of life (QoL) of patients. To explore the underlying pathophysiological mechanism, we investigated the correlation between the prefrontal cortex (PFC) function and OAB symptoms in PD patients.

**Methods:**

One hundred fifty-five idiopathic PD patients were recruited and classified either as PD-OAB or PD-NOAB candidates based on their corresponding OAB symptom scores (OABSS). A linear regression analysis identified a correlative connection of cognitive domains. Then cortical activation during the performance of the verbal fluency test (VFT) and brain connectivity during resting state were conducted by functional near-infrared spectroscopy (fNIRS) for 10 patients in each group to investigate their frontal cortical activation and network pattern.

**Results:**

In cognitive function analysis, a higher OABS score was significantly correlated with a lower FAB score, MoCA total score, and sub-scores of visuospatial/executive, attention, and orientation as well. In the fNIRS study, the PD-OAB group exhibited significant activations in 5 channels over the left hemisphere, 4 over the right hemisphere, and 1 in the median during the VFT process. In contrast, only 1 channel over the right hemisphere showed significant activation in the PD-NOAB group. The PD-OAB group revealed hyperactivation, particularly in certain channel in the left dorsolateral prefrontal cortex (DLPFC), compared with PD-NOAB (FDR *P* < 0.05). In the resting state, there was a significant increase of the resting state functional connectivity (RSFC) strength between the bilateral Broca area, left frontopolar area (FPA-L) and right Broca’s area (Broca-R), between the FPA and Broca’s area if merging the bilateral regions of interest (ROI), and also between the two hemispheres in the PD-OAB group. The Spearman’s correlation confirmed that the OABS scores were positively correlated with RSFC strength between the bilateral Broca area, FPA-L and Broca-R, between the FPA and Broca area if merging the bilateral ROI.

**Conclusion:**

In this PD cohort, OAB was related to decreased PFC functions, with particularly hyperactivated left DLPFC during VTF and an enhanced neural connectivity between the two hemispheres in the resting state as observed by fNIRS imaging.

## Introduction

Parkinson’s disease (PD) is caused by the gradual degeneration of the central nervous system (CNS) in elderly people and is characterized by four main motor symptoms, including resting tremor, rigidity, bradykinesia, and gaiting abnormalities. Recent findings have also recognized remarkable contributions of non-motor symptoms, like neuropsychiatric symptoms, autonomic dysfunctions, sleep disorders, pain, etc. Of these, lower urinary tract (LUT) dysfunction, predominately overactive bladder (OAB) with a prevalence rate of about 62%, becomes one of the most common types of autonomic disorders and seriously impacts the quality of life of PD patients and their caregivers (McDonald et al., 2017; Ogawa et al., 2017).

In addition to playing regulatory roles in micturition reflexes, the prefrontal cortex (PFC) directly modulates goal-directed and executive behaviors (Funahashi, 2001; Sakakibara et al., 2014). Recent investigations have highlighted that alteration of the D1 dopaminergic (DA) neuronal circuitry in the frontal-basal ganglion could be the major inducer of OAB in PD patients (Kitta et al., 2015; Mito et al., 2018). To date, only a few studies have elaborated on the pathological connection between PFC disturbances with OAB onset in PD from the neuropsychiatric perspective (Xu et al., 2019; Tkaczynska et al., 2020). However, there is still a lack of verification from functional neuroimaging examinations.

As the most widely used prefrontal lobe-related cognitive performance assessment method, the verbal (phoneme) fluency test (VFT) is often applied in the clinical evaluation of executive function-associated brain activation patterns, which might give clues to the underlying pathomechanisms in PD (Miller, 1985; Galtier et al., 2017). Functional near-infrared spectroscopy (fNIRS) at a wavelength range of 650∼950 nm is a relatively advanced non-invasive diagnostic modality of tracing changes in cerebral blood oxygen levels as indirect readouts of activities in cerebral cortices. It has the advantages of being capable of real-time monitoring, low-cost, safe and non-invasive, portable, etc. Compared with functional magnetic resonance imaging (fMRI), which is limited by the magnetic field and can only study “imaginary movement,” and electroencephalogram (EEG), which is easily interfered with motion artifacts, fNIRS can continuously and qualitatively collect brain function signals in dynamic movements and has been increasingly utilized in diagnosing and treating PD individuals (Zong et al., 2019; Mahmoudzadeh et al., 2021; Schejter-Margalit et al., 2022; Tao et al., 2023). Therefore, we attempted to evaluate the utility and efficacy of fNIRS in delineating the pathological correlation between OAB induction and PFC function in a PD sample to provide potential targets for future interventions.

## Materials and methods

### Subject selection

Between January 2021 and November 2022, 155 idiopathic PD subjects were enrolled from outpatients of neurology departments of Xinhua Hospital affiliated to Shanghai Jiao Tong University School of Medicine (XH-SJTUSM) and from the outpatients departments of neurology and neurosurgery of the first affiliated hospital of Navy Medical University (NMU) in this study. The Ethics Committee of respective hospitals approved the investigation, provided all participants agreed to give written informed consent. The patients were selected upon meeting the clinical diagnostic criteria of the Movement Disorder Society (MDS) and exhibiting positive responses to the levodopa therapy. If any subject was- (1) diagnosed with any non-PD neurological diseases and/or (2) severe urinary system diseases; (3) taking an anticholinergic drug, and (4) unable to communicate or coordinate with clinicians, he/she was promptly excluded from the study.

### Clinical assessments

Socio-demographic characteristics, including gender, age, education level, medical history, and disease duration were collected for the study subjects. The Hoehn-Yahr (H-Y) staging and MDS-Unified PD Rating Scale part III (MDS-UPDRS III) scores were used for evaluating the disease severity, while the Frontal Assessment Battery (FAB) and Montreal Cognitive Assessment (MoCA) scales were employed as cognitive performance evaluation. The psychopathological screening was done by the Hamilton Anxiety Rating Scale of 14 items (HAM-A) and the Hamilton Depression Rating Scale of 24 items (HAM-D). The patients’ OAB symptoms were evaluated by OABSS. The non-motor symptoms and QoL were evaluated through the NMSS and PD questionnaire 39 (PDQ39), respectively. All patients were evaluated in the on-medication states.

The PD patients were classified with or without OAB symptoms (PD-OAB, and PD-NOAB, respectively) based on the OABSS analysis, which has been a simple and effective method for scoring PD-specific symptoms: urgency, nighttime frequency, daytime frequency, and urge incontinence (Homma et al., 2006, 2011; Moussa et al., 2022). A higher OABSS score represents a worse condition. An OAB was diagnosed at OABSS ≥3, including an urgency score of ≥2.

### VFT, resting state, and fNIRS examinations

The VFT task was guided by a well-trained instructor. During the 30 s baseline period, subjects were required to sit on a chair and repeatedly go on counting from 1 to 16 until the task began. And throughout the 60 s task period, they were instructed to construct multiple phrases using simple words like big, earth, and sky. In the post-task recovery period, they were further asked to repeat counting from 1 through 32 till the end of the test.

In a resting state, subjects sit on a chair in a quiet room. They were asked to stay awake with their eyes closed, and not thinking about anything as much as possible.

**FIGURE 1:**
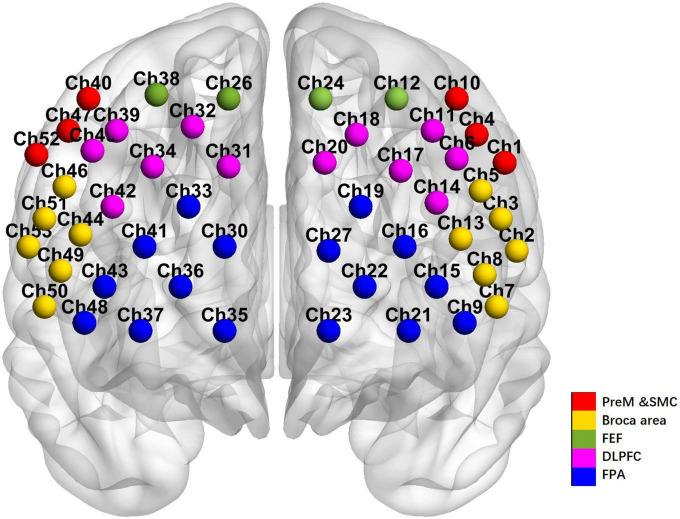
The arrangement of channels of the 53-channel near-infrared spectroscopy system according to the Brodmann’s map of the cortex.

A 53-channel BS-7000 fNIRS system (Wuhan Znion Technology Co., Wuhan, China) was utilized to longitudinally monitor the oxygenation changes (i.e., oxy- to deoxy-hemoglobin proportion) in the cerebral cortices throughout the VFT paradigm. A helmet containing numerous near-infrared light sensors covered both the prefrontal and temporal lobes of the subject. Distribution of near-infrared sensors followed the international standard of 10–20 leads, with the lowest channel positioned at Fp1–Fp2. The region of measurement covered the bilateral DLPFC (Channel 6, 11, 14, 17, 18, 20, 25, 31, 32, 34, 39, 42, 45), FPA (Channel 9, 15, 16, 19, 21, 22, 23, 27, 28, 29, 30, 33, 35, 36, 41, 43, 48), the Pre-Motor and Supplementary Motor Cortex (PreM and SMC) (Channel 1, 4, 10, 40, 47, 52), the Broca’s area (Channel 2, 3, 5, 7, 8, 13, 44, 46, 49, 50, 51, 53), and Frontal eye fields (FEF) (Channel 12, 24, 26, 38) according to the Brodmann map of the cortex (Figure 1). For VTF, the fNIRS assessment included a 30 s baseline, a 60 s VFT, and a 60 s post-task recovery period at a frequency of 20 Hz. For resting state, the fNIRS data collection lasted for 6 min for each subject.

### Statistical analysis

Continuous and categorical variables are, respectively, expressed as mean ± standard deviation (SD) and median (quartiles). A two-sample *t*-test comparing continuous variables while the chi-squared (χ^2^) comparing the categorical variables were performed between the PD-OAB and PD-NOAB groups. The Mann–Whitney U (MN-U) test was used to compare the differences in H-Y stages between the groups. A linear regression model was conducted according to OABSS with the enter method. Spearman’s analysis was used to discover the correlation between clinical variables and imaging features. *P*-values lesser than 0.05 were statistically significant. SPSS v25.0 was used for all these statistical analyses.

### fNIRS data analysis

All fNIRS data of brain networks were analyzed under the environment of Matlab R2014b (The Mathworks, United States) with Homer2_UI (Huppert et al., 2009) and NIRS-SPM software package^1^ (Ye et al., 2009) and visualized by NIRS_KIT^2^ (Hou et al., 2021) and BrainNet Viewer^3^ (Xia et al., 2013).

We mainly focus on oxy-Hb, in that oxy-Hb level reflects more directly cognitive activation and is more closely related to fMRI blood oxygenation level (Cui et al., 2011). For task-based data, the raw data were converted to changes in optical density using hmrIntensity2OD. HmrMotionArtifactByChannel and hmrmotioncorrectspline were applied to correct motion artifacts. HmrBandpassfilt (0.01–0.5 Hz) was applied to eliminate unrelated low- and high-frequency components. The optical density was then converted to changes in oxy-Hb concentration using hmrOD2Conc. The changes in oxy-Hb concentration in each channel on each individual was evaluated using a General Linear Model (GLM). The whole 30 s pre-task period were set as baseline and the task-period conditions were convolved with the standard typical hemodynamic response function (HRF) in the GLM model to form the corresponding regressors. The task-related β-value was determined as the β-value of the VFT minus the β-value of the baseline. The comparisons of β values between the two groups were conducted by a two-sample *t*-test. The false discovery rate (FDR) was utilized to correct for multiple comparisons (level set at *P* < 0.05) (Singh and Dan, 2006).

For the resting-state data, the first 10 s and the last 30 s of the data were dropped for a stable signal. Motion correction in preprocessing were the same as the above task-based processing. HmrBandpassfilt (0.01–0.1 Hz) was applied to eliminate unrelated components. Then, the functional matrix was obtained by calculating the Pearson correlation coefficient (*r*-value) of the time series of each channel. Fisher’s r-z transformation method was used to convert the obtained *r*-values to z-scores to create a 52 × 52 correlation matrix. For each group, a one-sample *t*-test was used to generate a group-specific RSFC map. RSFC strength between the ROI and two hemispheres for the PD-OAB and PD-NOAB groups was conducted by a two-sample *t*-test.

## Results

The mean age of recruited PD patients was 63.15 ± 10.05 years. This cohort had 64 (41.3%) female subjects. The clinical characteristics and comparisons between PD-OAB and PD-NOAB symptoms are shown in Table 1. PD-OAB patients were relatively older in age, had a longer disease duration, a higher LEDD, and advanced scores in H-Y staging, MDS-UPDRS-III, MoCA, FAB, PDQ39, and NMS scores. The gender distribution, years of education, and scores of HAM-A and HAM-D did not statistically differ between the groups.

**TABLE 1:** Comparisons of demographic and clinical features of PD subjects with or without OAB.

	Total sample (*n* = 155)	PD-OAB (*n* = 80)	PD-NOAB (*n* = 75)	*P*
Age (years), mean ± SD	63.15 ± 10.05	64.93 ± 9.51	61.49 ± 10.32	<0.05
Gender [n (female) (%)]	64 (41.3%)	36 (45%)	28 (37.3%)	0.333
Education (years), mean ± SD	12.13 ± 3.07	11.92 ± 2.79	12.32 ± 3.31	0.416
Disease duration (years), mean ± SD	7.88 ± 4.75	8.83 ± 4.46	7.00 ± 4.88	<0.05
Hoehn-Yahr staging, median (IQR)	3 (2.5, 3)	3 (3, 4)	3 (2, 3)	<0.05
MDS-UPDRS III	27.35 ± 14.47	30.25 ± 14.12	24.56 ± 14.35	<0.05
LEDD at admission (mg), mean ± SD	645.39 ± 473.15	754.32 ± 431.69	543.27 ± 489.91	<0.05
HAM-D, mean ± SD	12.09 ± 6.19	12.81 ± 5.71	11.41 ± 6.58	0.159
HAM-A, mean ± SD	10.00 ± 5.51	10.83 ± 5.40	9.22 ± 5.52	0.069
MoCA, mean ± SD	24.83 ± 2.93	24.03 ± 3.34	25.59 ± 2.24	<0.05
FAB, mean ± SD	16.14 ± 1.68	15.24 ± 1.48	16.98 ± 1.40	<0.05
PDQ-39, mean ± SD	61.01 ± 22.49	66.36 ± 21.17	56.00 ± 22.66	<0.05
NMSS, mean ± SD	17.35 ± 3.80	19.05 ± 3.41	15.73 ± 3.45	<0.05

MDS-UPDRS III, movement disorder society united Parkinson’s disease rating scale part III; LEDD, levodopa equivalent daily dosage; HAM-A, Hamilton Anxiety Rating Scale; HAM-D, Hamilton Depression Rating Scale; MoCA, Montreal Cognitive Assessment; FAB, Frontal Assessment Battery; PDQ39, Parkinson’s disease questionnaire 39; NMSS, non-motor symptoms scale; SD, standard deviation; IQR, interquartile range.

In cognitive function analysis, the linear regression exhibited that patients with higher OABSS scores presented significantly lower FAB scores, MoCA total scores, and sub-scores of visuospatial/executive, attention and orientation tests (Table 2).

**TABLE 2:** Linear regression analysis of cognitive performance with OABSS.

	PD-OAB	PD-NOAB	Standardized β	95% CI	*P*
MoCA total score	24.03 ± 3.35	25.59 ± 2.24	−0.25	−0.26∼−0.06	<0.05
Visuospatial/executive	2.94 ± 1.44	3.89 ± 0.96	−0.31	−0.68∼−0.22	<0.05
Naming	2.71 ± 0.49	2.77 ± 0.45	−0.09	−1.00∼0.30	0.29
Attention	5.68 ± 0.67	5.82 ± 0.38	−0.18	−1.20∼−0.08	<0.05
Language	2.51 ± 0.67	2.58 ± 0.63	−0.01	−0.52∼0.43	0.86
Abstraction	1.44 ± 0.60	1.63 ± 0.56	−0.14	−0.98∼0.06	0.08
Delayed recall	2.96 ± 1.52	2.95 ± 1.40	−0.01	−0.23∼0.20	0.89
Orientation	5.69 ± 0.60	5.90 ± 0.30	−0.20	−1.44∼−0.17	<0.05
FAB	15.24 ± 1.48	16.98 ± 1.40	−0.46	−0.68∼−0.36	<0.05

MoCA, Montreal Cognitive Assessment; FAB, frontal assessment battery.

**FIGURE 2:**
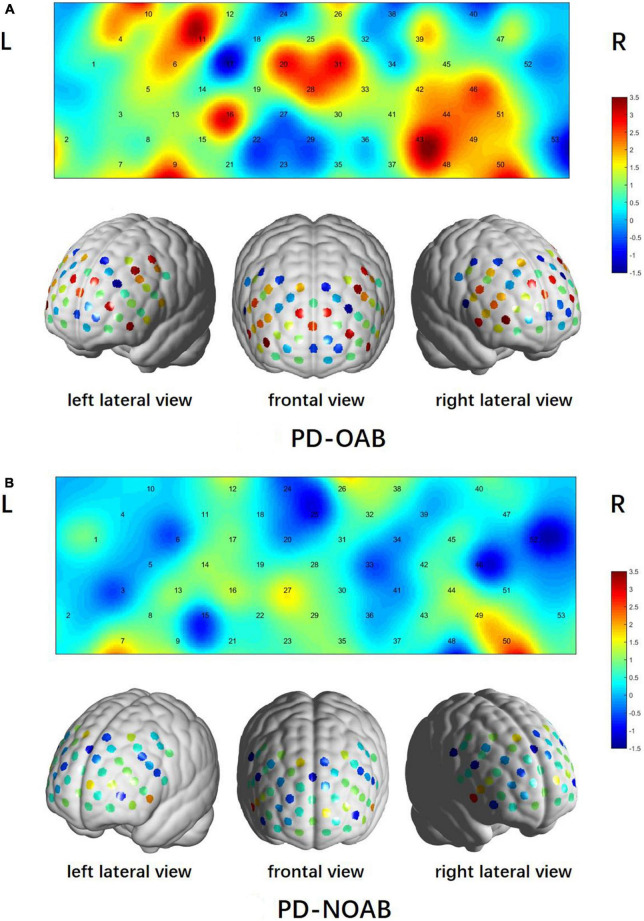
Patterns of cortical activations during VFT of PD-OAB **(A)** and PD-NOAB **(B)**.

**FIGURE 3:**
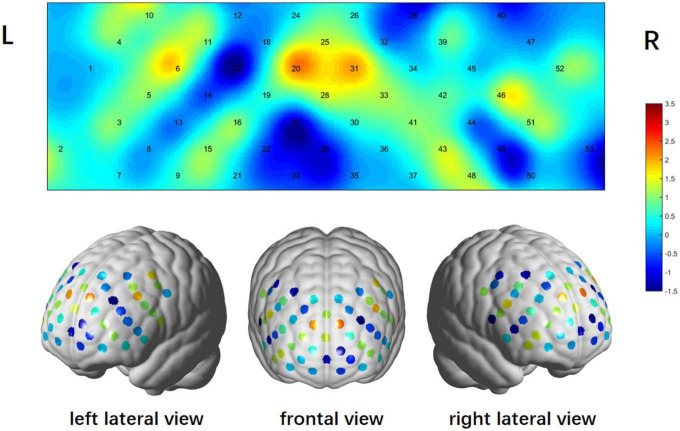
Differences in task-related cortical activations between the PD-OAB and PD-NOAB groups.

In the fNIRS study during VTF, the PD-OAB group revealed significant activations in 10 channels (9, 10, 11, 16, 20, 28, 31, 43, 46, and 50, *t* = 2.558–3.509, FDR *P* < 0.05) compared to the baseline condition, including 5 channels over the left hemisphere, 4 over the right hemisphere, and 1 in the median (Figure 2A). In contrast, only 1 channel (50, *t* = 2.792, FDR *P* < 0.05) over the right hemisphere showed significant activation compared to the baseline condition in the PD-NOAB group (Figure 2B). Moreover, compared with PD-NOAB group, the PD-OAB group demonstrated significant hyperactivation at channel 20 (*t* = 2.261, FDR *P* < 0.05) in the left DLPFC (Figure 3).

**FIGURE 4:**
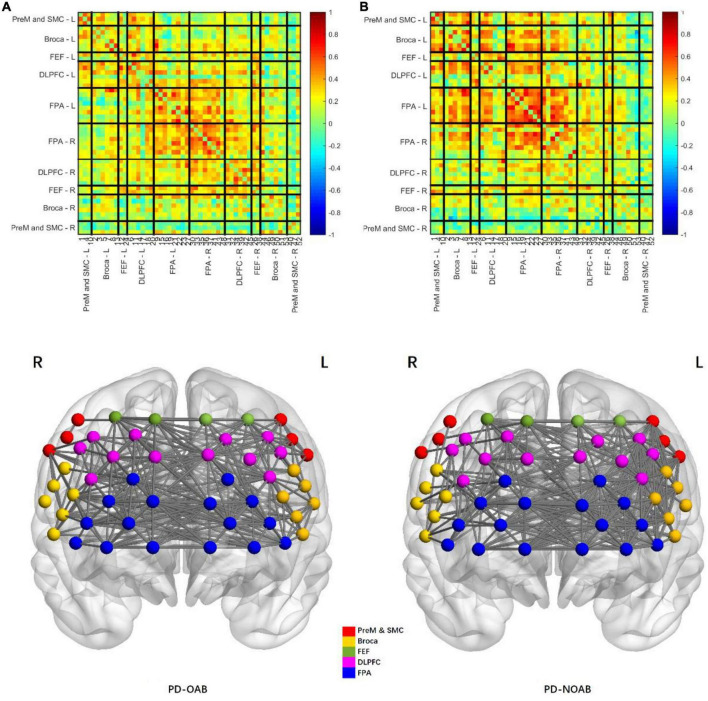
Pattern of resting-state functional connectivity strength between the regions of interest of PD-OAB **(A)** and PD-NOAB **(B)** groups.

**FIGURE 5:**
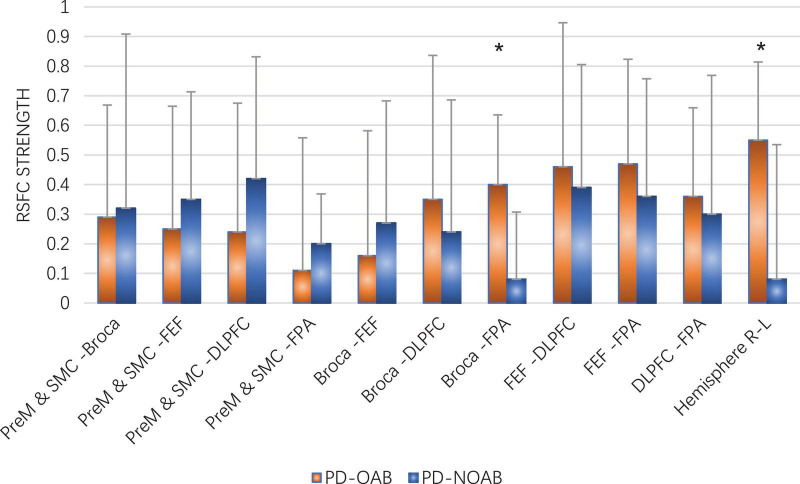
Two-sample *t*-test of RSFC strength between the ROI and two hemispheres for the PD-OAB and PD-NOAB groups. *P < 0.05.

The results of the RSFC pattern and two-sample *t*-test for the PD-OAB and PD-NOAB groups are presented in Figures 4, 5 and Supplementary Table 1. A two-sample *t*-test revealed that there was a significant increase in the RSFC strength between the bilateral Broca’s area (*P* = 0.027), FPA-L and Broca -R (*P* = 0.006), between the FPA and Broca’s area (*P* = 0.032) if merging the bilateral ROI, and also between the two hemispheres (*P* = 0.043) in the PD-OAB group.

Furthermore, the Spearman’s analysis revealed a positive correlation between the OABSS scores and FC strength of Broca R and L (*r* = 0.668, *P* = 0.013), FPA-L and Broca R (*r* = 0.779, *P* = 0.002), as well as Broca’s area to FPA if merging the bilateral ROI (*r* = 0.604, *P* = 0.029), but the correlation between OABSS scores and the FC of the two hemispheres or the activation strength (β value) at channel 20 yielded non-significant results.

## Discussion

Here, we investigated the association between OAB symptoms and PFC dysfunction in PD patients. The OABSS score was significantly correlated with lower FAB/MoCA total scores and sub-scores of visuospatial/executive, attention and orientation. In the fNIRS study, the PD-OAB group showed a hyperactivation particularly at channel 20 in the left DLPFC during VTF and an enhanced neural connectivity between the two hemispheres in the resting state compared with that in the PD-NOAB, suggesting a possibility of the underlying link between these two symptoms.

Our data showed that PD patients with higher OABSS presented significantly lower FAB scores and MoCA total scores, especially in visuospatial/executive, attention and orientation-related domains, which was well-aligned with previous reports. In a cohort of 189 non-demented PD subjects, urinary urgency was found to be associated with executive dysfunctions, in combination with NMS burden, nocturia, and stigma (Tkaczynska et al., 2020). Xu et al. (2019) have also identified that H-Y staging, FAB scoring, and RBD were accompanying etiological factors for OAB. There could be a multitude of pathological connections underlying the cross-talks between OAB and PFC symptoms, which gradually appear and progress along with the disease severity, probably due to the spread of α-synuclein plaques to the diencephalon and neocortex, corresponding to Braak stages 4–5 in PD (Braak et al., 2003). The urinary tract symptoms of PD patients may also be caused by disruption of the neurotransmitter (e.g., glutamate, dopamine, serotonin, and norepinephrine) signaling networks (Buddhala et al., 2015). A decline in nigrostriatal DAergic function has been linked to urinary tract pathologies in PD, as confirmed by functional neuroimaging studies (Winge et al., 2005; Mito et al., 2018; Wang et al., 2020). Besides, given the older ages of these subjects, the influence of cerebrovascular burden couldn’t be eliminated in linking OAB to cognition (Buchman et al., 2011).

Many studies have explored PD with LUT from the perspective of imaging. An MRI study suggests that the thickness and volume of the precuneus (left) and left frontal pole may manifest the severity of LUT symptoms in PD patients (Roh et al., 2021). Kitta et al. (2006) have found significant brain activations in the periaqueductal gray, cerebellar vermis, supplementary motor area, putamen, insula, and thalamus during detrusor overactivity with positron emission tomography (PET) scanning in nine male PD patients. A ^123^I-FP-CIT SPECT study involving 31 untreated PD patients indicated PD-OAB individuals had lower striatal DAT availabilities (Mito et al., 2018). An alteration in brain raphe nuclei positioning was reported by Walter et al. (2006) in a cohort of PD-OAB patients using transcranial sonography. Despite the utilization of a variety of research instruments, there is no definite conclusion on the location of the damage and the underlying pathophysiological mechanism of PD with OAB. Hence, strict inclusion criteria, a combination of multiple research methods, relatively large sample sizes, and longitudinal follow-ups may help obtain reliable conclusions in the future.

To our knowledge, no current publications have investigated the cognition function in PD patients with OAB by using fNIRS. Our data revealed that certain PFC regions were activated during VFT and an enhanced neural connectivity between the two hemispheres in the resting state in the PD-OAB group than that in the PD-NOAB group, suggesting this subset of patients may require additional cognitive support to compensate for the damaged bladder control and PFC could be a better target in improving the intervention strategy under this condition. Cortex-specific non-invasive brain stimulation could be a potential approach to evaluate changes in cognition. Our results were in line with several previous findings showing higher brain activation in the PFC of PD patients with a freeze of gait (FoG) than that in PD patients without FoG, indicating a positive correlation between clinical severities and cortical activation strengths (Belluscio et al., 2019; Pu et al., 2022). However, we could not detect any statistical significance in this correlation between the clinical severity with cortical activation, which might be due to some confounding factors between the two that have not been identified and adjusted. Or, the correlation between the two may be not linear, by reason of the undurability of the compensatory response of the prefrontal cortex in the advanced stage of the disease. With these results, we plan further to carry out a cohort study with intervention on ROI using a sample size sufficient for stratified analysis.

The major strength of this study was the good generalizability of the conclusion. This study highlighted the outpatient PD subpopulation complicated with OAB symptoms and the possible diagnostic and interventional strategies. Other strengths included the advantages of combining the neuropsychological battery with advanced imaging modalities like fNIRS in the precise evaluation of cortical hemodynamic changes in PD. The crucial limitations of this study were: first, we couldn’t explore the causative factors that linked OAB to cognitive impairment because of the cross-sectional nature of this study. Future longitudinal studies will be useful in unveiling the pathological role of neuronal circuitry loss in PFC-inducing OAB symptoms. Second, urodynamic examination reports weren’t available for stratifying PD-OAB subjects from PD-NOAB. Despite our strict exclusion criteria, it was difficult to completely avoid false positives and false negatives. Finally, unlike fMRI, fNIRS measurements exhibited a moderate spatial resolution and might not be suitable for investigating the relationship between cortical and subcortical structures. The fNIRS, nevertheless, could provide an overall evaluation of neuronal activities in the PFC.

## Conclusion

Non-motor symptoms of OAB, secondary to PD, can seriously impact the quality of life in PD patients and their caregivers. Our study demonstrated that decreased PFC function could be associated with OAB in PD by using neuropsychological examinations and that the pattern of PFC activation and network measured by task-based and resting-state fNIRS altered in PD patients with OAB. The findings of this study are likely to improve our understanding of non-motor complications like OAB in PD patients and highlight a potential alternative intervention target.

## Data availability statement

The raw data supporting the conclusions of this article will be made available by the authors, without undue reservation.

## Ethics statement

The studies involving human participants were reviewed and approved by the Ethics Committees of the Xinhua Hospital Affiliated to Shanghai Jiao Tong University School of Medicine and the First Affiliated Hospital of Naval Medical University. The patients/participants provided their written informed consent to participate in this study.

## Author contributions

JG and ZGL designed the research. XM, JW, YZ, CQ, LS, YiW, and ZHL examined the patients and recruited the data. YaW performed the statistical analysis. MH drafted the manuscript. All authors made contributions to this study, critically reviewed the content, and approved the final version of this article.

## Footnotes

1
https://www.nitrc.org/projects/nirs_spm/

2
https://doi.org/10.1117/1.NPh.8.1.010802

3
http://www.nitrc.org/projects/bnv/
