# Supplementary material for: Pattern of prefrontal cortical activation and network revealed by task-based and resting-state fNIRS in Parkinson’s disease’s patients with overactive bladder symptoms
Source: Front Neurosci. 2023 Mar 30;17:1142741. doi: 10.3389/fnins.2023.1142741 (PMC10098071; doi:10.3389/fnins.2023.1142741)
Supplement: Supplementary file 2 [file Table_2.DOCX]

**Supplementary table 1** two sample t-test for RSFC strength between ROI and the two hemispheres of PD-OAB and PD-NOAB groups.

| Group name | PD-OAB | | PD-NOAB | | P |
| --- | --- | --- | --- | --- | --- |
|  | Mean | SD | Mean | SD |  |
| ROI to ROI |  |  |  |  |  |
| PreM & SMC - L -Broca - L | 0.38 | 0.32 | 0.61 | 0.26 | 0.19 |
| PreM & SMC - L -FEF - L | 0.23 | 0.55 | 0.25 | 0.63 | 0.97 |
| PreM & SMC - L -DLPFC - L | 0.54 | 0.33 | 0.38 | 0.53 | 0.51 |
| PreM & SMC - L -FPA - L | 0.13 | 0.29 | 0.46 | 0.42 | 0.12 |
| PreM & SMC - L -FPA - R | 0.20 | 0.24 | 0.25 | 0.39 | 0.76 |
| PreM & SMC - L -DLPFC - R | 0.30 | 0.38 | 0.20 | 0.44 | 0.67 |
| PreM & SMC - L -FEF - R | 0.24 | 0.38 | 0.55 | 0.26 | 0.11 |
| PreM & SMC - L -Broca - R | -0.02 | 0.44 | -0.13 | 0.53 | 0.70 |
| PreM & SMC - L -PreM & SMC - R | -0.12 | 0.42 | 0.03 | 0.39 | 0.50 |
| Broca - L -FEF - L | 0.27 | 0.51 | 0.12 | 0.54 | 0.59 |
| Broca - L -DLPFC - L | 0.39 | 0.41 | 0.40 | 0.60 | 0.95 |
| Broca - L -FPA - L | 0.39 | 0.36 | 0.59 | 0.28 | 0.31 |
| Broca - L -FPA - R | 0.42 | 0.38 | 0.21 | 0.51 | 0.43 |
| Broca - L -DLPFC - R | 0.28 | 0.26 | 0.14 | 0.36 | 0.45 |
| Broca - L -FEF - R | 0.28 | 0.30 | 0.27 | 0.31 | 0.98 |
| Broca - L -Broca - R | 0.30 | 0.30 | -0.33 | 0.50 | 0.03 |
| Broca - L -PreM & SMC - R | 0.11 | 0.40 | -0.07 | 0.55 | 0.51 |
| FEF - L -DLPFC - L | 0.40 | 0.62 | 0.58 | 0.27 | 0.51 |
| FEF - L -FPA - L | 0.29 | 0.29 | 0.32 | 0.56 | 0.88 |
| FEF - L -FPA - R | 0.42 | 0.20 | 0.37 | 0.42 | 0.79 |
| FEF - L -DLPFC - R | 0.16 | 0.46 | 0.45 | 0.31 | 0.20 |
| FEF - L -FEF - R | 0.42 | 0.37 | 0.29 | 0.56 | 0.63 |
| FEF - L -Broca - R | -0.05 | 0.47 | 0.11 | 0.51 | 0.56 |
| FEF - L -PreM & SMC - R | 0.01 | 0.38 | 0.01 | 0.39 | 0.99 |
| DLPFC - L -FPA - L | 0.29 | 0.32 | 0.20 | 0.58 | 0.72 |
| DLPFC - L -FPA - R | 0.20 | 0.35 | 0.33 | 0.37 | 0.54 |
| DLPFC - L -DLPFC - R | 0.49 | 0.33 | 0.20 | 0.34 | 0.15 |
| DLPFC - L -FEF - R | 0.37 | 0.40 | 0.16 | 0.38 | 0.34 |
| DLPFC - L -Broca - R | 0.18 | 0.45 | 0.08 | 0.49 | 0.71 |
| DLPFC - L -PreM & SMC - R | -0.04 | 0.31 | 0.19 | 0.35 | 0.23 |
| FPA - L -FPA - R | 0.68 | 0.16 | 0.53 | 0.49 | 0.44 |
| FPA - L -DLPFC - R | 0.34 | 0.41 | 0.34 | 0.46 | 0.98 |
| FPA - L -FEF - R | 0.38 | 0.45 | 0.47 | 0.35 | 0.72 |
| FPA - L -Broca - R | 0.43 | 0.18 | -0.26 | 0.40 | 0.01 |
| FPA - L -PreM & SMC - R | -0.01 | 0.45 | -0.18 | 0.27 | 0.45 |
| FPA - R -DLPFC - R | 0.44 | 0.28 | 0.48 | 0.39 | 0.82 |
| FPA - R -FEF - R | 0.46 | 0.33 | 0.28 | 0.39 | 0.39 |
| FPA - R -Broca - R | 0.25 | 0.35 | 0.12 | 0.37 | 0.53 |
| FPA - R -PreM & SMC - R | -0.07 | 0.52 | 0.31 | 0.35 | 0.16 |
| DLPFC - R -FEF - R | 0.38 | 0.45 | 0.31 | 0.56 | 0.81 |
| DLPFC - R -Broca - R | 0.26 | 0.47 | 0.04 | 0.34 | 0.37 |
| DLPFC - R -PreM & SMC - R | -0.18 | 0.53 | 0.12 | 0.28 | 0.25 |
| FEF - R -Broca - R | 0.11 | 0.41 | 0.07 | 0.42 | 0.86 |
| FEF - R -PreM & SMC - R | 0.03 | 0.46 | -0.10 | 0.34 | 0.58 |
| Broca - R -PreM & SMC - R | 0.03 | 0.43 | 0.39 | 0.59 | 0.23 |
| ROI to ROI  (left and right merged) |  |  |  |  |  |
| PreM & SMC -Broca | 0.29 | 0.38 | 0.32 | 0.59 | 0.93 |
| PreM & SMC -FEF | 0.25 | 0.41 | 0.35 | 0.36 | 0.66 |
| PreM & SMC -DLPFC | 0.24 | 0.43 | 0.42 | 0.41 | 0.47 |
| PreM & SMC -FPA | 0.11 | 0.45 | 0.20 | 0.17 | 0.63 |
| Broca -FEF | 0.16 | 0.42 | 0.27 | 0.41 | 0.64 |
| Broca -DLPFC | 0.35 | 0.49 | 0.24 | 0.45 | 0.66 |
| Broca -FPA | 0.40 | 0.24 | 0.08 | 0.23 | 0.03 |
| FEF -DLPFC | 0.46 | 0.49 | 0.39 | 0.42 | 0.78 |
| FEF -FPA | 0.47 | 0.35 | 0.36 | 0.40 | 0.61 |
| DLPFC -FPA | 0.36 | 0.30 | 0.30 | 0.47 | 0.80 |
| Hemisphere R-L | 0.55 | 0.26 | 0.08 | 0.45 | 0.04 |

Abbreviations: ROI, region of interest; FPA, left frontopolar area; DLPFC, dorsolateral prefrontal cortex; FEF, frontal eye fields; PreM & SMC, Pre-Motor and Supplementary Motor Cortex.
